# Inadvertent Bowel Injury Following Repeated Suprapubic Catheterisation in a Patient of Post-Pelvic Radiotherapy

**DOI:** 10.7759/cureus.10189

**Published:** 2020-09-01

**Authors:** Shikhar Verma, Sivaranjit K Vaka, Ankit Jain, Abhinaya Reddy, Vishnu Prasad Nelamangala Ramakrishnaiah

**Affiliations:** 1 Surgery, Jawaharlal Institute of Postgraduate Medical Education and Research, Puducherry, IND

**Keywords:** suprapubic catheterisation, bowel injury, pelvic radiation

## Abstract

Suprapubic catheterization (SPC) is one of the standard procedures in urological emergencies. The common complications of SPC include loss of track, hematuria, catheter blockage, and catheter-related infections. However, severe complications like bowel injuries, including intestinal obstructionand perforation, can also occur.

We present the case of a 54-year-old lady who had received pelvic radiation 30 years ago for carcinoma cervix. She presented to a secondary-level care center with anuria. On failure of per urethral catheterization, she repeatedly underwent unguided SPC. However, unsatisfied with her recovery, she was brought to our tertiary care center by her relatives. She was found to have inadvertent placement of SPC in the small bowel, which was confirmed preoperatively by ultrasound and CT. Intraoperatively, the SPC catheter was seen inside the terminal ileum causing ileal wall necrosis and a localized feco-purulent collection. Urinary bladder rent was also noted at the site of the earlier SPC. Resection of distal ileum with double barrel ileostomy, followed by primary repair of the bladder wall, was done. Unfortunately, she succumbed to overwhelming sepsis and expired in the postoperative period. This case emphasizes a potential higher risk of life-threatening bowel injury due to SPC insertion in patients with previous pelvic irradiation. Such high-risk cases should be approached with the utmost care, preferably under ultrasound guidance. For safe practice, the British Association of Urological Surgeons’ guidelines for SPC insertion should be followed.

## Introduction

Suprapubic catheterization (SPC) is one of the standard procedures in urological emergencies. The procedure is considered simple, safe, and lifesaving in some instances. The procedure is safer when it is done under the guidance of the ultrasound or a flexible cystoscopy by an experienced urologist [1]. However, the life-threatening complication of bowel injury following SPC is always on the surgeon’s mind, as it carries significant morbidity and mortality [2]. Bowel perforation can present at any time after SPC insertion, even years later, and in few cases, might not present with abdominal pain [3].

## Case presentation

A 54-year-old lady, known case of chronic kidney disease, presented to a secondary-level care center with anuria. She had received pelvic radiation 30 years ago for carcinoma cervix. An attempt of per urethral catheterization failed, and thus non-guided SPC was done, which drained thick pus. Once the urine output improved and the infection was resolved, SPC was clamped to see if she could pass per urethra, which she passed well. Subsequently, SPC was removed after four weeks. Fifteen days following this, she started having purulent discharge from the previous SPC site. On visiting the same hospital, SPC was reinserted through the previous SPC site but was accidentally pulled out on the 10th day of re-insertion. SPC re-insertion through the previous site failed, so the SPC was inserted 1 cm proximal to the old SPC site. Again, the procedure was done unguided. Post-SPC, her urine output did not improve, and some particulate material was also noticed in the SPC tube. Not satisfied with the recovery of the patient, relatives brought the patient to our tertiary care center.

On presentation, the patient only had complaints of mild lower abdomen pain with decreased urine output. There was no history of fever, nausea/vomiting, or non-passage of flatus/feces. On examination, there was tachycardia of 110/min, Blood pressure was 100/70 mmHg, and the respiratory rate was 15/min. On abdominal examination, lower abdomen tenderness was noted, but there were no signs of peritonitis. There was the fecal matter at the SPC site and in the SPC drainage bag. Digital rectal examination was unremarkable with normal fecal staining. Per vaginal examination did not reveal any growth. Based on the above findings, a clinical diagnosis of enterovesical fistula, most probably rectovesical, was made.

**Figure 1:**
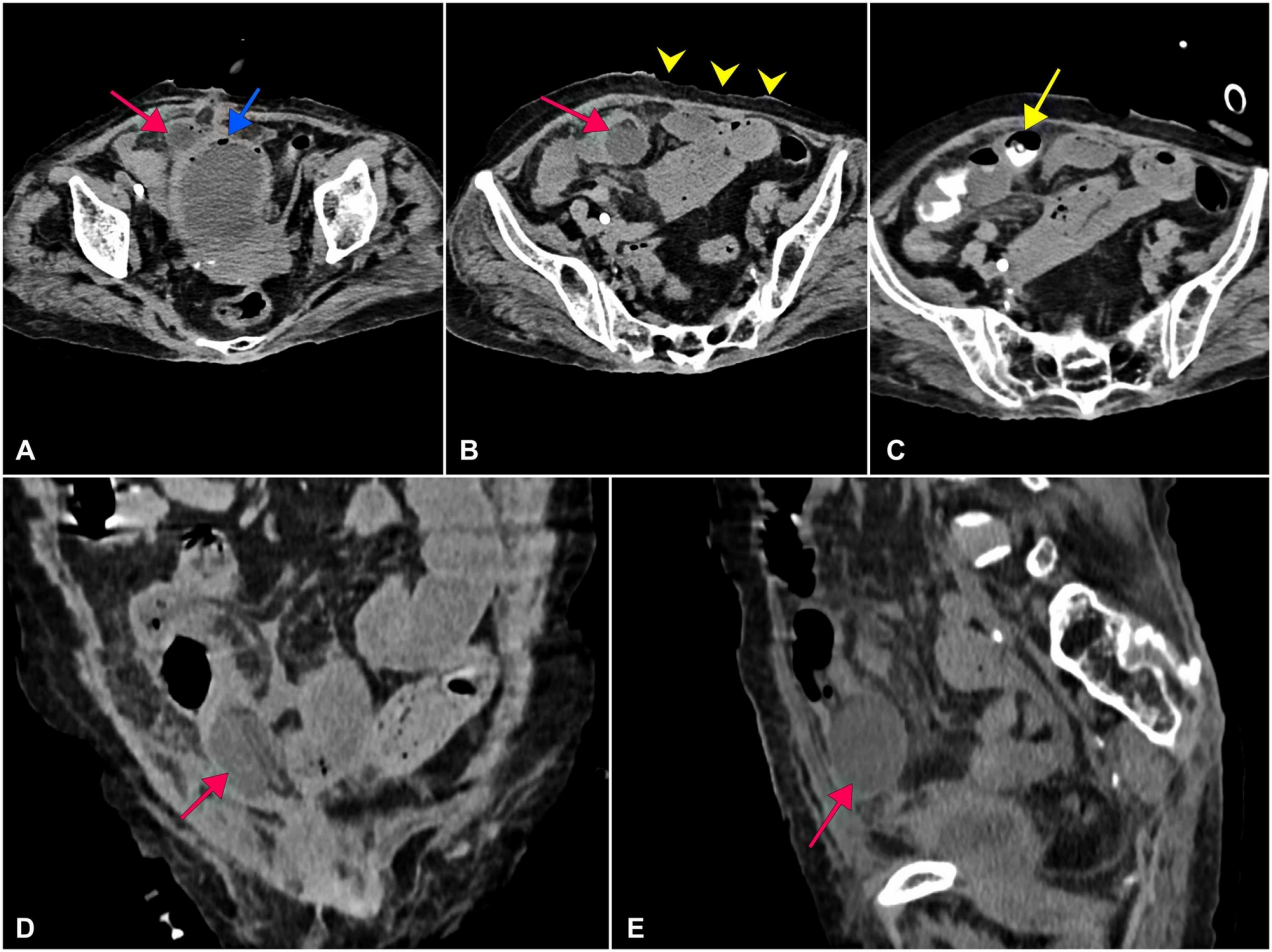
CT images showing the Foley bulb in the ileal loop (red arrows). The blue arrow shows the distended bladder with air specks. The yellow arrowheads show the radiation changes in the skin and adhered bowel loops just underneath the abdominal wall. The yellow arrow shows contrast flowing from the tip of the Foley catheter into the ileal loops. (A-C) Axial sections. (D) Coronal section. (E) Sagittal section.

Her blood investigation revealed leukocytosis of 33,000/mm^3^ (4,000-11,000/mm^3^) with acute kidney injury with blood urea 123 mg/dL (15-40 mg/dL), serum creatinine 4 mg/dL (0.6-1.2 mg/dL), serum sodium 126 mEq/L (136-146 mEq/L), and serum potassium 5.7 mEq/L (3.5-5.0 mEq/L). There was no metabolic acidosis. Ultrasonography revealed a distended bladder with moving echoes. The Foley bulb was seen outside the bladder with the surrounding collection and air foci. She was evaluated with CT of the abdomen, which showed Foley bulb in the distal ileum but without pneumoperitoneum, suggestive of a sealed perforation. On instilling contrast through the Foleys, the contrast was seen in the ileal loops without leakage inside the peritoneal cavity, thus confirming the diagnosis (Figure 1). She was resuscitated and started on broad-spectrum antibiotics. Per urethral catheterization was done, and 200 mL of pyuria was drained. Because of acute kidney injury, one session of hemodialysis was done preoperatively. The patient was taken up for emergency exploratory laparotomy.

**Figure 2:**
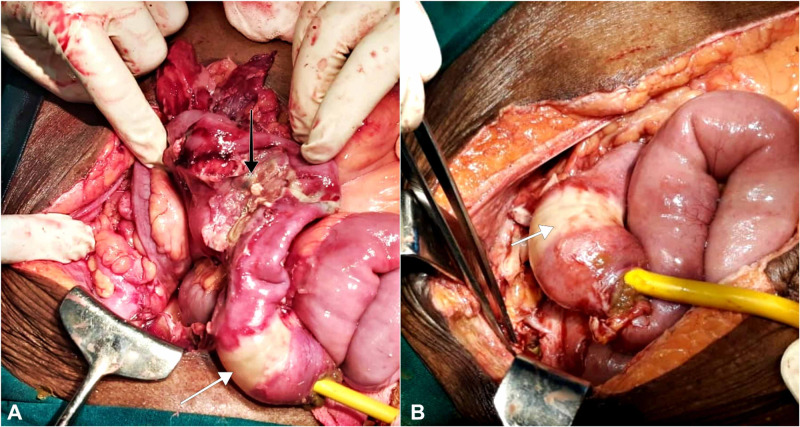
(A, B) Intraoperative images showing the Foley catheter in the ileal loop with demarcated necrotic changes (white arrow) around the region of the Foley bulb and gangrenous distal ileum (black arrow). Surrounding ileal loops are seen inflamed with pus flakes.

Intraoperatively, the SPC catheter was seen inside the terminal ileum, around 30 cm from the ileocecal junction. There was ileal wall necrosis at the site of the catheter bulb. Approximately 10 cm distal to it, a 2-cm segment of distal ileum was sloughed off with localized feco-purulent collection (50 mL) (Figure 2). Urinary bladder rent of 2 cm was noted at the site of the earlier SPC. Resection of distal ileum with double barrel ileostomy, followed by primary repair of the bladder wall with re-insertion of the SPC under direct vision, was done. Unfortunately, she succumbed to overwhelming sepsis and expired in the postoperative period. 

## Discussion

Percutaneous SPC is a simple and effective procedure for draining the bladder in acute urinary retention cases when urethral access is not feasible [4], in trauma patients with urethral injury, urinary incontinence, and spinal diseases [5]. Contraindications to SPC include bladder carcinoma, bleeding diathesis, abdominal wall infection, and a subcutaneous vascular graft in the suprapubic region [6]. Techniques commonly used for SPC are direct blind trocar puncture, image (ultrasonography/cystoscopy/fluoroscopy) guided trocar placement, or open surgical approach. For a safe procedure, British Association of Urological Surgeons’ (BAUS) guidelines published in 2011 made the following recommendations [6]: the bladder should be adequately distended with 300 mL of distilled water or saline, the patient is to be placed in a Trendelenburg position, the bladder should be palpable at least 5 cm above the pubic symphysis [7], and the catheter should pass through the rectus sheath in the midline, not more than 2 cm above the pubic symphysis.

Often considered to be a simple procedure, it is usually done by the trainee surgeons. If not done carefully, it can lead to various complications [8]. The common complications include loss of track (misplacement), hematuria, catheter blockage, and catheter-related infections. However, severe complications like bowel injuries, including intestinal obstruction and perforation, can also occur [9]. Bowel perforation can occur at any time, during tube insertion or even during catheter exchange [10]. Conditions predisposing to such an injury are neuropathic bladder, chronic inflammatory states like diverticulitis and pelvic inflammatory disease, history of previous abdominal surgeries, or history of pelvic irradiation, like in the current case. 

Although the earlier series reported a 2%-3% rate of bowel injuries [2,5], a recent audit from the UK on 11,473 patients revealed an improving trend with rates ranging from 0% to 0.2% [11]. This improvement is because of the National Patient Safety Agency (NPSA) report published in 2009 and BAUS guidelines published in 2011, which recommended ultrasound-guided insertion in all patients having high-risk features, as stated above, however only by a practitioner with appropriate training and experience [6,12]. Ultrasound was also recommended when the bladder cannot be palpated despite adequate filling, such as in obese patients. There has been no large published data proving the safety profile of ultrasound-guided catheter insertions. However, many small series and the experience of ultrasound-guided central venous catheter placements suggest its use strongly, especially in high-risk groups [13]. It is also recommended that in patients with previous lower abdominal surgery or other high-risk factors, or a bladder that will not distend, an open SPC approach should be used using a small suprapubic incision [6,8,12]. The open approach ensures the catheter track does not injure the bowel, which may be adhered to the abdominal wall or bladder.

## Conclusions

This case emphasizes that although a potential risk of life-threatening bowel injury is inherent to the SPC procedure, it is higher in patients with previous pelvic irradiation. Such high-risk cases should be approached with the utmost care, preferably under ultrasound guidance. However, the safest method is an open cystotomy whereby a careful dissection helps to visualize the presence of bowel loops and prevent injury. Also, a high suspicion for such complications should be kept in mind so that timely corrective measures can be administered. For safe practice, we advocate a universal adoption of the BAUS' guidelines for SPC insertion.
